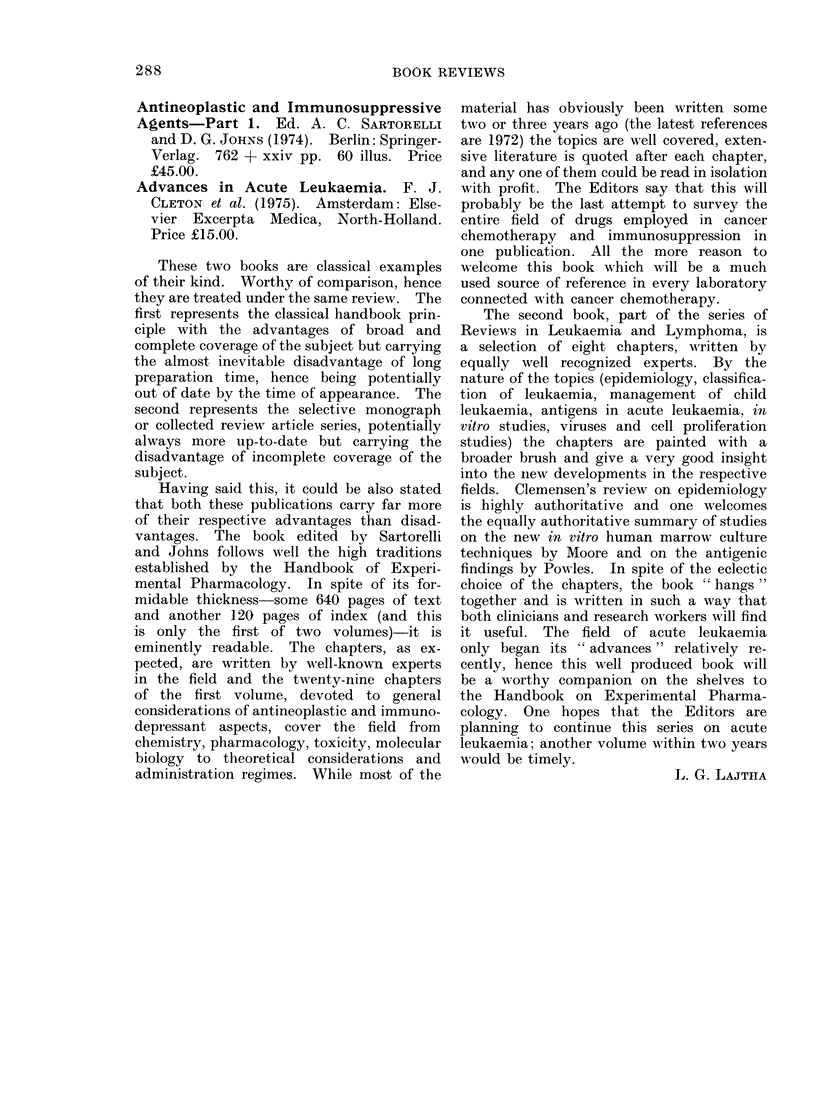# Antineoplastic and Immunosuppressive Agents—Part 1

**Published:** 1975-08

**Authors:** L. G. Lajtha


**Antineoplastic and Immunosuppressive Agents—Part 1.** Ed. A. C. Sartorelli and D. G. Johns (1974). Berlin: Springer-Verlag. 762 + xxiv pp. 60 illus. Price £45.00.

**Advances in Acute Leukaemia.** F. J. Cleton *et al.* (1975). Amsterdam: Elsevier Excerpta Medica, North-Holland. Price £15.00.

These two books are classical examples of their kind. Worthy of comparison, hence they are treated under the same review. The first represents the classical handbook principle with the advantages of broad and complete coverage of the subject but carrying the almost inevitable disadvantage of long preparation time, hence being potentially out of date by the time of appearance. The second represents the selective monograph or collected review article series, potentially always more up-to-date but carrying the disadvantage of incomplete coverage of the subject.

Having said this, it could be also stated that both these publications carry far more of their respective advantages than disadvantages. The book edited by Sartorelli and Johns follows well the high traditions established by the Handbook of Experimental Pharmacology. In spite of its formidable thickness—some 640 pages of text and another 120 pages of index (and this is only the first of two volumes)—it is eminently readable. The chapters, as expected, are written by well-known experts in the field and the twenty-nine chapters of the first volume, devoted to general considerations of antineoplastic and immunodepressant aspects, cover the field from chemistry, pharmacology, toxicity, molecular biology to theoretical considerations and administration regimes. While most of the material has obviously been written some two or three years ago (the latest references are 1972) the topics are well covered, extensive literature is quoted after each chapter, and any one of them could be read in isolation with profit. The Editors say that this will probably be the last attempt to survey the entire field of drugs employed in cancer chemotherapy and immunosuppression in one publication. All the more reason to welcome this book which will be a much used source of reference in every laboratory connected with cancer chemotherapy.

The second book, part of the series of Reviews in Leukaemia and Lymphoma, is a selection of eight chapters, written by equally well recognized experts. By the nature of the topics (epidemiology, classification of leukaemia, management of child leukaemia, antigens in acute leukaemia, *in vitro* studies, viruses and cell proliferation studies) the chapters are painted with a broader brush and give a very good insight into the new developments in the respective fields. Clemensen's review on epidemiology is highly authoritative and one welcomes the equally authoritative summary of studies on the new *in vitro* human marrow culture techniques by Moore and on the antigenic findings by Powles. In spite of the eclectic choice of the chapters, the book " hangs " together and is written in such a way that both clinicians and research workers will find it useful. The field of acute leukaemia only began its " advances " relatively recently, hence this well produced book will be a worthy companion on the shelves to the Handbook on Experimental Pharmacology. One hopes that the Editors are planning to continue this series on acute leukaemia; another volume within two years would be timely.

L. G. Lajtha